# Endoscopic variceal ligation combined with endoscopic submucosal dissection in the treatment of esophageal varices complicated by early esophageal cancer

**DOI:** 10.1055/a-2414-7539

**Published:** 2024-10-25

**Authors:** Huan Ma, Yuan-jing He, Li-meng Wu, Xin-hua Zhao, Xiao-an Li

**Affiliations:** 1 Department of Gastroenterology, Mianyang Central Hospital, School of Medicine, University of Electronic Science and Technology of China, Mianyang, China; 2 Department of Burn and Plastic Surgery, Mianyang Central Hospital, School of Medicine, University of Electronic Science and Technology of China, Mianyang, China

A 61-year-old patient with cirrhosis and esophageal varices underwent gastroscopy, during which mucosal lesions, about 3 cm in length and 1.5 cm in width, were found in the lower esophagus ( Fig. 1 ). Pathological examination revealed high grade intraepithelial neoplasia, and endoscopic submucosal dissection (ESD) treatment was planned. However, the right posterior wall of the lesion was closely related to a varicose vein, and the whole local area was located on the edge of the vein, which could not be avoided during mucosal incision. Therefore, endoscopic variceal ligation (EVL) was first used to ligate the distal end of the varicose vein to block blood flow, and then ESD was performed to treat the mucosal lesions ( Video 1 ).

**Fig. 1:**
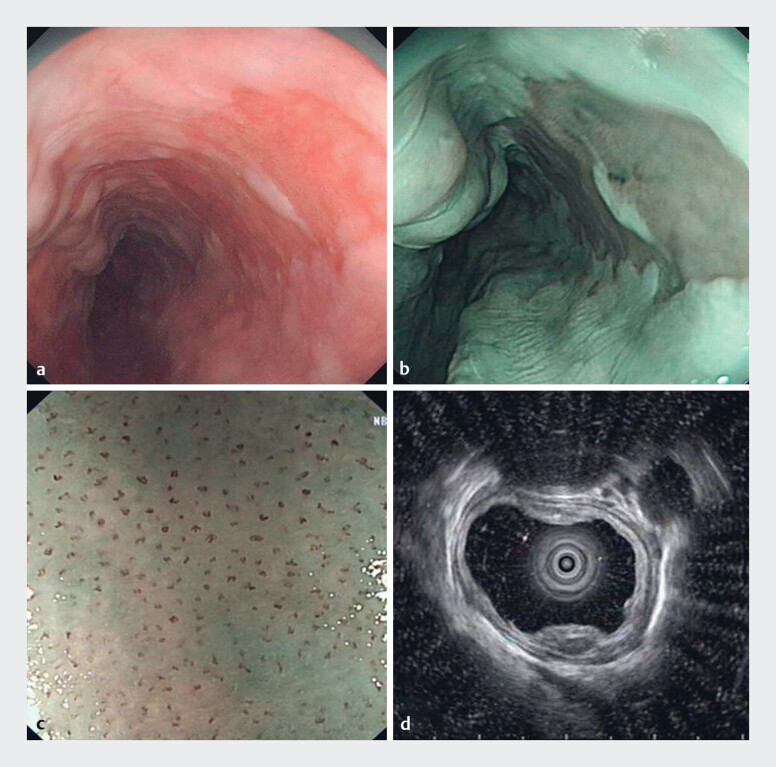
Preoperative imaging. **a** Endoscopy showed a red section of mucous membrane at 30–33 cm from the incisors. **b** The lesions under narrow-band imaging were slightly brown. **c** Magnifying endoscopy showed dilation and distortion of intraepithelial papillary capillary loop. **d** Endoscopic ultrasonography showed that the local mucosa was hypoechoic and slightly thickened, and the submucosa, lamina propria, and adventitia were of normal appearance.

A new technique for treating esophageal varices complicated by early esophageal cancer.Video 1

Intraoperative blood loss was small and the field of vision was clear. Postoperative pathology confirmed high grade intraepithelial neoplasia with negative horizontal and vertical resection margins ( Fig. 2 ). The patient was followed up for 1 month, 3 months, 6 months, and 12 months, without bleeding, dysphagia, and other discomfort.

**Fig. 2:**
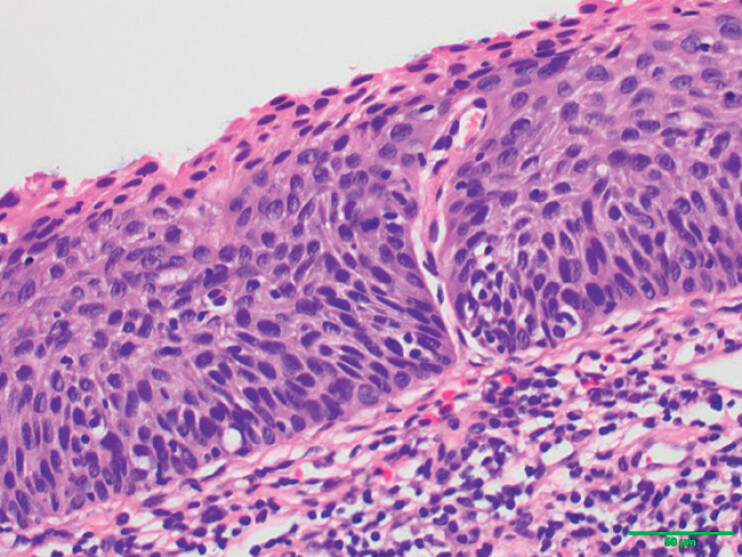
Postoperative pathology confirmed high grade mucosal intraepithelial neoplasia.

Owing to the existence of thick varices, esophageal cancer is easy to overlook in the early stages in patients with liver cirrhosis complicated by esophagogastric varices 1 . Moreover, they often have a significantly increased surgery risk due to poor coagulation function and high risk of bleeding. In the past, some centers have tried endoscopic mucosal resection for the treatment of esophageal varices combined with superficial early esophageal cancer 2 3 4 5 . In the current case, we successfully treated the lesions of esophageal varices combined with early esophageal cancer through EVL and ESD, and there was no recurrence for half a year and 1 year after the operation. This case indicated that such treatment is feasible in patients with liver cirrhosis and esophageal varices combined with early esophageal cancer.

Endoscopy_UCTN_Code_TTT_1AO_2AG_3AD

## Notes

Endoscopy E-Videos https://eref.thieme.de/e-videos
*E-Videos* is an open access online section of the journal *Endoscopy* , reporting on interesting cases and new techniques in gastroenterological endoscopy. All papers include a high-quality video and are published with a Creative Commons CC-BY license. Endoscopy E-Videos qualify for HINARI discounts and waivers and eligibility is automatically checked during the submission process. We grant 100% waivers to articles whose corresponding authors are based in Group A countries and 50% waivers to those who are based in Group B countries as classified by Research4Life (see: https://www.research4life.org/access/eligibility/ ). This section has its own submission website at https://mc.manuscriptcentral.com/e-videos .
